# Supplementary material for: Fracture Characteristics of Commercial PEEK Dental Crowns: Combining the Effects of Aging Time and TiO2 Content
Source: Polymers (Basel). 2023 Jun 17;15(12):2720. doi: 10.3390/polym15122720 (PMC10303836; doi:10.3390/polym15122720)
Supplement: Supplementary file 1 [file polymers-15-02720-s001.zip › polymers-2402342-supplementary.pdf]

Supplementary Materials

Fracture Characteristics of Commercial PEEK Dental Crowns: Combining the Effects of Aging Time and TiO_2 Content

by Wen-Ju Lu, Wei-Cheng Chen, Viritpon Srimaneepong, Chiang-Sang Chen, Chang-Hung Huang, Hui-Ching Lin, Oi-Hong Tung and Her-Hsiung Huang

1. Glass transition region analysis

Differential scanning calorimetry (DSC) (DSC 4000, PerkinElmer, Shelton, USA) analysis was performed on the test PEEK crowns with and without 10-h aging treatment, including BreW (no aging), BreW (10-h aging), BreA (no aging), and BreA (10-h aging), to estimate whether the glass transition region (Tg approximately 151 °C [14]) was different among the test groups. The test sample weighing approximately 10 mg was encapsulated in a sealed aluminum pan, heated with a temperature ramp of 6 °C/min from room temperature to about 200 ◦C, then cooled from 200 °C to room temperature under nitrogen flow of 20 mL/min. Each analysis was performed twice.

Figure S1 presents the DSC analysis results, showing no significant difference in the glass transition region among the test groups. This implied that the 10-h aging and TiO_2 content (20 or 30 %) did not have significant effect on the glass transition region of commercial dental PEEK crowns used in this study.

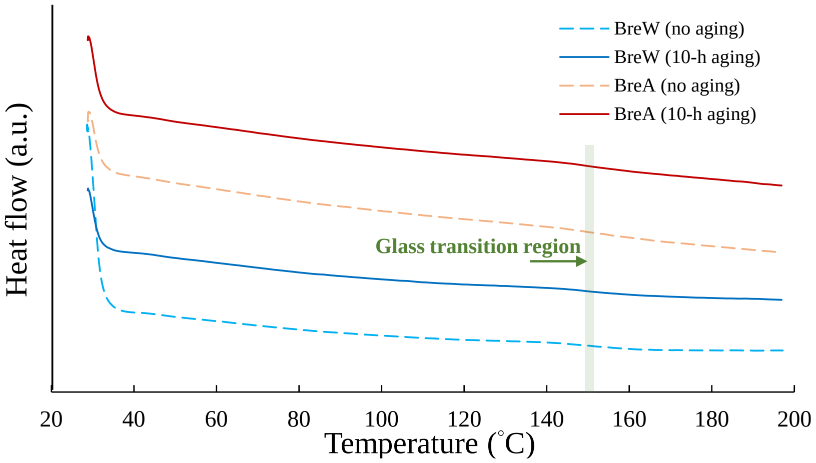

Figure S1. DSC curves (second heating) for the test PEEK crowns, showing no significant difference in the glass transition region among the test four groups.
